# Evaluation of the mechanical properties and degradation behavior of chitosan-PVA-graphene oxide nanocomposite scaffolds in vitro

**DOI:** 10.1016/j.jtumed.2024.04.008

**Published:** 2024-04-30

**Authors:** Asmaa M. Ali, Sonia M. Elshabrawy, Elbadawy A. Kamoun

**Affiliations:** a Dental Biomaterial Department, Faculty of Dentistry, Kafr El-Sheikh University, Egypt; b Dental Biomaterials Department, Faculty of Dentistry, Alexandria University, Alexandria, Egypt; c Department of Chemistry, College of Science, King Faisal University, Al-Ahsa, KSA; d Polymeric Materials Research Department, Advanced Technology and New Materials Research Institute, City of Scientific Research and Technological Applications, Alexandria, Egypt

**Keywords:** الكيتوسان, البولي فينيل الكحول, جزيئات أكسيد الجرافين النانوية, الخصائص الميكانيكية, التحلل البيولوجي, Biodegradation, Chitosan, Graphene oxide nanoparticles, Mechanical properties, Polyvinyl alcohol

## Abstract

**Objectives:**

Chitosan (CTS) has been a popular option for scaffold fabrication because of its biocompatibility, biodegradability, antimicrobial and nonimmunogenic effects. However, it is of limited function, due to its low mechanical strength and its solubility in acidified media. These limitations could be overcome by its blending with PVA and incorporation with bioactive materials to improve its mechanical properties and tissue regeneration capability.

**Methods:**

Carbon based nanomaterials, such as graphene oxide (GO) incorporated with CTS/PVA blend to improve composite-scaffold stability. GO nanoparticles were chemically prepared and fully characterized. Different concentrations of both CTS and nano-GO were used for the fabrication of CTS/PVA/GO nanocomposite films through the solvent-casting method. The mechanical properties, thermal stability biodegradation, and swelling of the nanocomposite films were evaluated after characterization by XRD, FTIR and SEM, to detect the effect of GO incorporation in the scaffold to select the suitable dental application.

**Results:**

A better performance was observed in thermal stability, biodegradation, and water resistance after GO addition into CTS/PVA scaffolds. Regarding mechanical properties, groups were assessed by *Kruskal Wallis* test afterward Dunn’s post hoc test. There was no significant difference in tensile strength between the nanocomposite films of CTS (2%) and CTS (3%). The tensile strength decreased after addition of nano-GO at different concentrations. The elastic modulus significantly increased when (1%) GO was added into the 1CTS (2%):1PVA.

**Conclusions:**

CTS/PVA/GO nanocomposite can be used in dental hard tissue engineering, as the incorporation of GO into the CTS/PVA polymer blend improves its properties which is regarded as the critical concentrations of CTS and GO.

## الملخص

**أهداف البحث:**

يعتبر الكيتوسان مادة شائعة لتصنيع السقالات بسبب توافقها الحيوي، وقابليتها للتحلل البيولوجي، والتأثيرات المضادة للجراثيم وعدم المناعية. ومع ذلك، فهو ذو وظيفة محدودة بسبب ضعف قوتها الميكانيكية وذوبانها في الوسائط المحمضة. يمكن التغلب على هذه القيود من خلال خلطها مع البولي فينيل الكحول وإدماجها مع المواد النشطة حيويا لتحسين خصائصها الميكانيكية وقدرتها على تجديد الأنسجة.

**طرق البحث:**

تم إدماج المواد النانوية المستندة إلى الكربون، مثل أكسيد الجرافين، مع خليط الكيتوسان والبولي فينيل الكحول لتحسين ثبات سقالة البناء المركب. تم إعداد جزيئات أكسيد الجرافين النانوية كيميائيا وتوصيفها بالكامل. تم استخدام تركيزات مختلفة من كل من الكيتوسان والجرافين النانوي لتصنيع أفلام مركبة نانوية من الكيتوسان والبولي فينيل الكحول وأكسيد الجرافين من خلال طريقة الصب المذيب. تم تقييم الخصائص الميكانيكية والاستقرار الحراري والتحلل البيولوجي والانتفاخ للأفلام المركبة النانوية بعد التوصيف باستخدام تحليل حيود الأشعة السينية والمطياف تحت الحمراء والمجهر الإلكتروني الماسح، لاكتشاف تأثير إدماج أكسيد الجرافين في السقالة لاختيار التطبيق السني المناسب.

**النتائج:**

لوحظ أداء أفضل في الاستقرار الحراري والتحلل البيولوجي ومقاومة الماء بعد إضافة أكسيد الجرافين إلى سقالات الكيتوسان والبولي فينيل الكحول. فيما يتعلق بالخصائص الميكانيكية، تمت تقييم المجموعات باستخدام اختبار كروسكال واليس ثم اختبار دون البعدي. لم يكن هناك فرق كبير في قوة الشد بين أفلام المركبات النانوية من الكيتوسان بتركيز 2% والكيتوسان بتركيز 3%. انخفضت قوة الشد بعد إضافة أكسيد الجرافين النانوي بتركيزات مختلفة. ازداد معامل المرونة بشكل كبير عند إضافة 1% من أكسيد الجرافين إلى خليط الكيتوسان بتركيز 2% والبولي فينيل الكحول.

**الاستنتاجات:**

يمكن استخدام المركب النانوي من الكيتوسان والبولي فينيل الكحول وأكسيد الجرافين في هندسة أنسجة الأسنان الصلبة، حيث أن إدماج أكسيد الجرافين في مزيج بوليمر الكيتوسان والبولي فينيل الكحول يحسن خصائصه مع مراعاة التركيزات الحرجة من الكيتوسان وأكسيد الجرافين.

## Introduction

The human body is a complex structural system with multiple types of cells and tissues. Researchers are studying the applications of multiple biomaterials to repair defects in the body.^1^, ^2^, ^3^ Tissue engineering is aimed at regenerating damaged tissues through biologically compatible techniques. The main components of tissue engineering are cells, growth factors, and scaffolds.^1^ Optimization of scaffolds can create ideal conditions for cellular growth. For effective use, scaffolds must meet certain criteria, including sufficient biocompatibility, porosity, degradation rate, mechanical strength, sterility, and cost-effectiveness.^4^ Both organic and synthetic polymer-based biomaterials are used for scaffold construction. Natural polymers including collagen, gelatin, chitosan (CTS), cellulose, and alginate, are biocompatible agents that can increase cellular adhesion and proliferation. However, they lack the mechanical strength necessary for some applications, such as hard tissue engineering.^5^ Polyvinyl alcohol (PVA) is considered a favorable candidate for tissue regeneration, because of its controlled biodegradability, but it has several disadvantages, such as poor migration of metabolites and stimulation of inflammatory reactions during its degradation. In general, degradable polymers have very low mechanical properties, whereas mechanically strong polymers are always bioinert.^6^^,^^7^

CTS-based biomaterials have received substantial attention and are widely used in a variety of applications, because of their specific characteristics, including low foreign body reactions, antibacterial activity, biodegradability, and biocompatibility.^8^ CTS can form porous structures—a key characteristic in tissue engineering and cell transplantation. CTS is derived from chitin, which is present in the exoskeletons of crustaceans.^9^ This linear, pseudo-natural, and semi-crystalline polysaccharide is composed of (1 → 4)-2-acetamido-2-deoxy-β-D-glucan (N-acetyl d-glucosamine) and (1 → 4)-2-amino-2-deoxy-β-D-glucan (d-glucosamine).^10^ However, chitin is difficult to use in biomedical applications because it is insoluble in water. Consequently, chitin is deacetylated and transformed to CTS. Enzymatic or alkaline deacetylation removes the acetyl group from the C-2 position of chitin, thereby resulting in insertion of a primary amine group. Chitin becomes soluble in acidic media when its acetylation level exceeds 50%, because of protonation of the amine group.^10^^,^^11^ The primary amine group, which has a positive charge and is responsible for CTS's ability to interact with negatively charged macromolecules, such as DNA, RNA, and other biological components, is also responsible for CTS's antibacterial activity, muco-adhesiveness, and hemostatic properties.^12^^,^^13^ CTS, like many polysaccharides, has several inherent disadvantages, such as poor solubility and stability in physiological fluids, and insufficient flexibility; consequently, it is rarely used in its pure form and instead is combined with other materials.^14^

PVA is a synthetic water-soluble polymer with exceptional biocompatibility, nontoxicity, and non-carcinogenicity; thus, it is considered an ideal biomaterial for use in polymer blends.^15^^,^^16^ PVA also can increase the flexibility of other polymers.^16^ The CTS/PVA polymer blend becomes non-miscible at high concentrations of PVA (>50%). Miscibility occurs only at lower PVA concentrations, because of PVA's weak intermolecular attraction and strong intramolecular structure.^17^ CTS/PVA blends are used for multiple medical purposes, e.g., tissue engineering,^18^ wound dressings,^19^ and drug delivery systems.^20^ Recently, numerous inorganic substances, including clays and ceramic particles, have been applied as reinforcing agents to modify the final polymer's characteristics and performance.^21^ Additionally, carbon-based nanomaterials, including carbon nanotubes, graphene, and graphene oxide (GO), are currently used because of their exceptional mechanical, optical, electrical, and biological properties.^22^ GO nanosheets provide a biocompatible platform for cellular growth and tissue regeneration.^23^ These materials have also received substantial interest in a variety of biomedical applications, including drug delivery,^24^ bioimaging,^25^ tissue engineering,^26^ and antibacterial treatments.^27^ GO can be prepared through the modified Hummer method with a high product yield.^28^

Because GO has oxygen-containing functional groups, such as epoxy, carboxyl, and hydroxyl groups, on its nanosheet basal plane and edge, it is more hydrophilic and dispersible in water than graphene and graphite powder.^29^^,^^30^ These functional groups engage in strong interactions with polar solvents and polymer matrices, thus facilitating GO dispersion.^31^ GO is also effective against Gram-positive and Gram-negative bacteria.^32^, ^33^, ^34^ This antibacterial action occurs through various mechanisms, including high oxidative stress, membrane stress and cell wrapping; GO has also been found to promote cell proliferation in a dose-dependent manner.^35^^,^^36^ Graphene-based coatings have been reported to protect implants against corrosion by preventing bacterial colonization.^37^

GO positively influences the proliferation and differentiation of mesenchymal stem cells, as reported in some in vitro cultures.^38^^,^^39^ Moreover, GO has the necessary properties for the development of biosensors, including good electrical conductivity, large surface area, and high electromechanical activity.^40^ Biopolymer-GO applications have been reported, given that GO is considered nontoxic and can improve polymer properties even at very low concentrations, in contrast to other reinforcing fillers.^41^

Herein, we focused on evaluating the mechanical properties, biodegradation behavior, water sorption, and thermal stability of CTS-PVA-GO nanocomposites with different ratios of CTS and GO in the scaffold composition. The null hypothesis was that no difference would be observed in the mechanical properties, biodegradation, water sorption, and thermal stability of different concentrations of CTS and GO in a nanocomposite scaffold.

## Materials and Methods

### Study design

In this in vitro study, 136 samples were used for characterization, and the evaluation of mechanical properties, biodegradation, and water sorption. The sample size was calculated on the basis of a 95% confidence level and 80% study power with Rosner's method^42^ in *G∗Power* software version 3.0.10 (*G∗Power* 2019, Universität Düsseldorf, Germany). The study samples were divided into eight groups according to the tested concentrations of CTS (2% or 3%) and GO (0%, 0.3%, 0.5%, and 1%). Each group comprised 17 samples: five samples for characterization, eight samples for evaluation of mechanical properties, two samples for biodegradation assessment, and two samples for swelling measurement.

### Materials and Methods

Sulfuric acid (purity 95–97%) was purchased from Riedel deHaen (Germany), hydrogen peroxide (purity ∼36%) was purchased from Pharaohs Trading Co. (Egypt), hydrochloric acid (30%) was purchased from El-Salam for Chemical Industries (Egypt), potassium permanganate (99%) was obtained from Long-live Co. (China), graphite (200 nm mesh, 99.99%) was obtained from Alpha-Aesar (Germany), and CTS powder (moderate molecular weight, 75–85% deacetylated) and PVA (hydrolysis degree, 98–99%; molecular weight, 31–50 KDa) were obtained from Sigma–Aldrich (Saint Louis, MO, USA). Phosphate buffered saline (PBS) and acetic acid were supplied by El Goumhoria Co. for Chemicals (Egypt).

### Preparation of GO nanoparticles

GO was synthesized through a slightly modified Hummer method, as previously reported.^43^ Graphite powder (3 g) was added to 70 ml concentrated H_2_SO_4_, and the mixture was stirred with a mechanical stirrer for 10 min while being kept in an ice bath and monitored with a thermometer to regulate the temperature. Over 1 h, 9.0 g KMnO_4_ was gradually integrated into the suspension and stirred at room temperature. Subsequently, 5.0 g potassium persulfate was gradually added to the suspension solution. To prevent a sudden rise in reaction temperature, an ice bath and thermometer were used to maintain a constant temperature of 25 °C. After 1 h, the suspension solution was added to 500 ml distilled water, and 15 ml hydrogen peroxide was subsequently added. The resulting solid was filtered and rinsed once with double-distilled water, once with acidic water (10% HCl), and twice with double-distilled water. The resultant substance was dried at 70 °C for 48 h, then stored for subsequent experiments.^43^

### Characterization of GO nanoparticles

GO was characterized through the following methods: Raman spectroscopy, UV/visible spectrophotometry (double beam model T80+, PG instruments Ltd., UK), FTIR (BRUKER, Germany) covering the spectrum range of 400–4000 cm^−1^, X-ray diffraction analysis (XRD) (XRD-7000 Shimadzu, Japan), SEM, and EDX (JEOL, JSM-5 investigator model, Japan).

### Preparation of CTS/PVA/GO nanocomposite films

Two concentrations of CTS were prepared (2% and 3% [wt./v]) by dissolving CTS in 2% acetic acid under stirring for 5 h at room temperature.^44^ Subsequently, 10% PVA solution was prepared in distilled water at 60 °C with stirring for 2 h. The polymer blends were obtained by mixing two polymeric solutions (50% CTS:50% PVA), as reported elsewhere.^45^ In a solution containing 2% acetic acid, GO was dispersed at three concentrations (0.3%, 0.5%, and 1% [wt./v]). Sonication was used to ensure uniform distribution of the mixture.^44^^,^^45^ CTS/PVA/GO nanocomposite films were obtained through the solvent-casting method. GO solution was added to the polymer blend and stirred for 24 h until a homogeneous mixture was obtained. The solution was transferred into a Petri dish, and the casted film was dried in a vacuum oven at 60 °C overnight.^44^

### Sample groups

Samples were assigned to two main groups and one control group according to the CTS concentration in the nanocomposite. Each main group was divided into the following three subgroups according to the concentration of GO nanoparticles in the nanocomposite:

Group I, control group (CTS only): subgroup IA, CTS 2% (wt./v); subgroup IB, CTS 3% (wt./v)
Group II, CTS 2% (wt./v): subgroup IIA, GO 0.3% (wt./v); subgroup IIB, GO 0.5% (wt./v); subgroup IIC, GO 1% (wt./v)
Group III, CTS 3% (wt./v): subgroup IIIA, GO 0.3% (wt./v); subgroup IIIB, GO 0.5% (wt./v); subgroup IIIC, GO 1% (wt./v)

### Instrumental characterization of prepared CTS/PVA/GO nanocomposite films

The thermal behavior of the nanocomposites was evaluated through thermal gravimetric analysis (TGA). Thermal stability was evaluated with TGA/DCS (Shimadzu, Japan) under a nitrogen atmosphere. Evaluation of the mechanical properties of the nanocomposite was conducted through two modes as follows. (a) Tensile strength tests were conducted according to ISO 2062:2009 specifications. The extension rate was kept at 5 mm/min, and the cell load was fixed at 20 N with a gauge length of 30 mm.^44^ The dimensions of the samples were 6 cm × 1 cm.^46^ The tensile strength was evaluated for both CTS/PVA and CTS/PVA/GO nanocomposite films (Fig. S1, *supplementary data*). (b) In Young's modulus evaluation, the elastic modulus in both CTS/PVA and CTS/PVA/GO nanocomposite films was evaluated to assess the effect of GO incorporation on the scaffold.

### In vitro biodegradation assessment

Samples of CTS/PVA and CTS/PVA/GO nanocomposite films (10 × 10 mm) were placed in 5 ml PBS (pH = 7.4) to evaluate the biodegradation rate. The samples were weighed and then stored in an incubator at 37 °C. After 7, 14, 21, and 28 days, samples were removed from the PBS, rinsed in double-distilled water, dried in a 40 °C oven, and weighed again. Final calculations of the weight loss percentage (WL) were performed according to the following equation:

(1) WL = [(W0–W1)/W0] × 100%

where W0 is the weight of the sample before soaking, and W1 is the weight of the sample after soaking.^44^

### Swelling study

Gravimetric measurements were conducted to assess the films' swelling kinetics under ambient conditions. The films were fully dried in a vacuum oven, and their weight (Wi) was determined; they were then placed in a beaker containing 50 ml saline. After removal of surface water with a filter paper, the difference in the film's weight (Wt.) was measured after 1, 3, 24, and 48 h with the following equation^14^:

(2) Swelling ratio (%) = [(Wt.- Wi)/Wi] × 100.

### Statistical analysis

Variables are presented as median, interquartile range (IQR), minimum, and maximum values, in addition to mean and standard deviation (SD). Variables were compared with the Kruskal Wallis test followed by Dunn's post hoc test with Bonferroni correction. The significance level was set at a *P* value of 0.05. Data were analyzed in IBM SPSS (version 23.0).

### Abbreviations

CTS, Chitosan; PVA, Polyvinyl alcohol; and GO, Graphene oxide.

## Results

### Characterization of GO nanosheets

#### Raman spectroscopy

**Figure 1:**
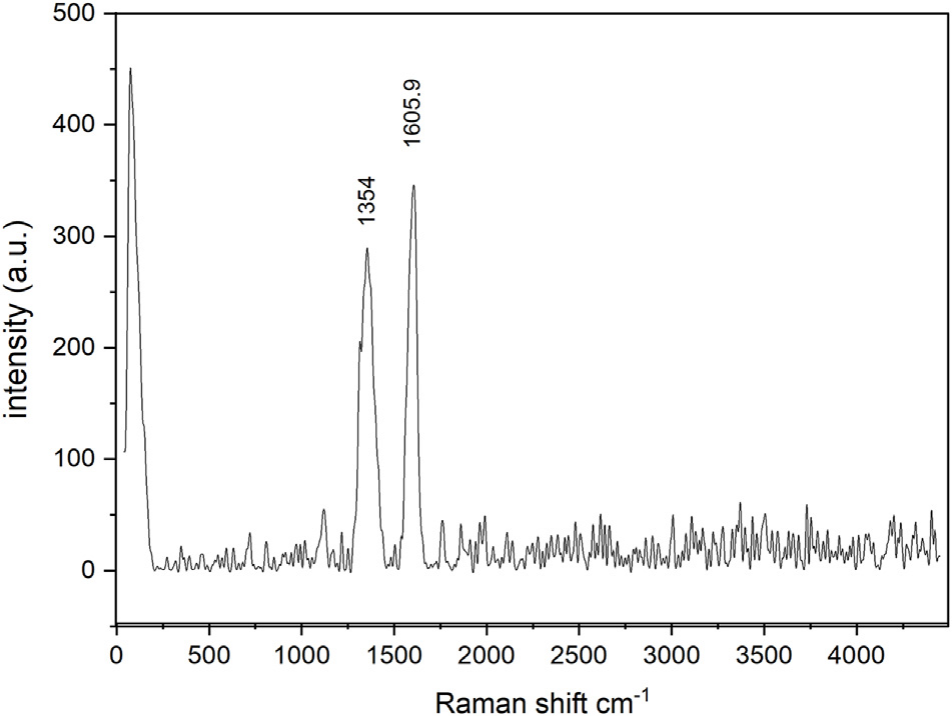
Raman spectroscopy of graphene oxide nanoparticles.

In the Raman spectra of GO (Figure 1), the G band was broad, and shifted to ν 1605 cm^−1^. The D band had high intensity because of disordering in the *sp*^*2*^ structure and was observed at ν 1354 cm^−1^.

#### FTIR analysis

**Figure 2:**
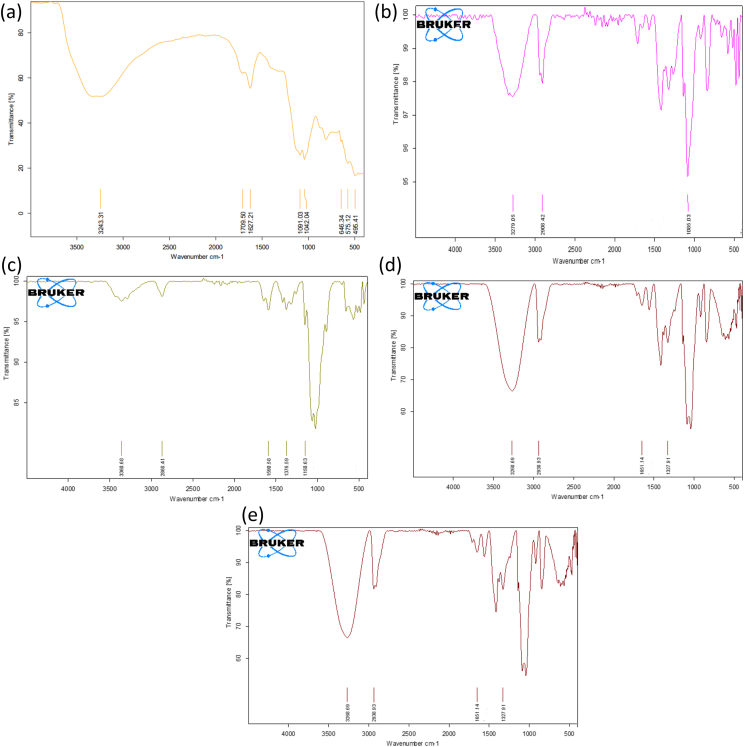
FTIR spectra of (a) GO, (b) PVA, (c) CTS, (d) CTS/PVA blend, and (e) CTS/PVA/GO.

The spectrum of GO (Figure 2a) exhibited multiple characteristic peaks corresponding to multiple oxygen-containing functional groups, thus confirming the formation of GO nanosheets. Bands at approximately ν 1709 cm^−1^, ν 1091–1042 cm^−1^, and ν 3243 cm^−1^ corresponded to C=O (carboxyl or carbonyl), C–O (epoxy or alkoxy), and O–H stretching of the COOH group, respectively. The peak around ν 811 cm^−1^ was attributed to aromatic C–H deformation. Spikes at ν 646, 575, and 495 cm^−1^ were attributed to C–H bending vibration. The band at ν 1627 cm^−1^ was associated with the remaining *sp*^*2*^ structure.

#### XRD analysis

**Figure 3:**
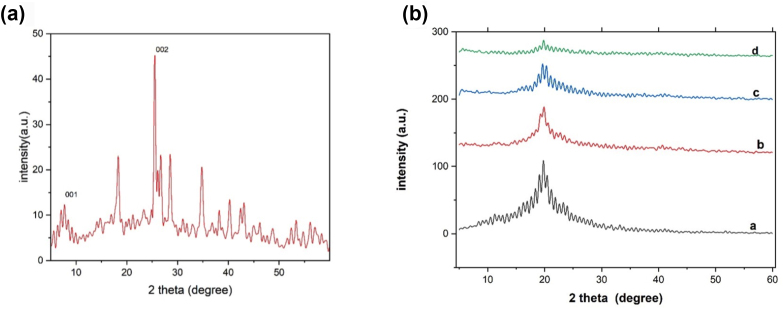
(a) XRD patterns of GO powder. (b) CTS/PVA blend, blend with 0.3% GO, blend with 0.5% GO, and blend with 1% GO.

The spectrum of the prepared GO (Figure 3a) exhibited a characteristic diffraction pattern at 2θ of 8°, which corresponded to an interlayer distance of the (001) plane of approximately 12.4 Å. Another diffraction peak observed at 26.9° corresponded to the (002) plane and d spacing of 3.4 Å. This peak appeared because of the remaining graphitic *sp*^*2*^ structure.

#### SEM investigation

**Figure 4:**
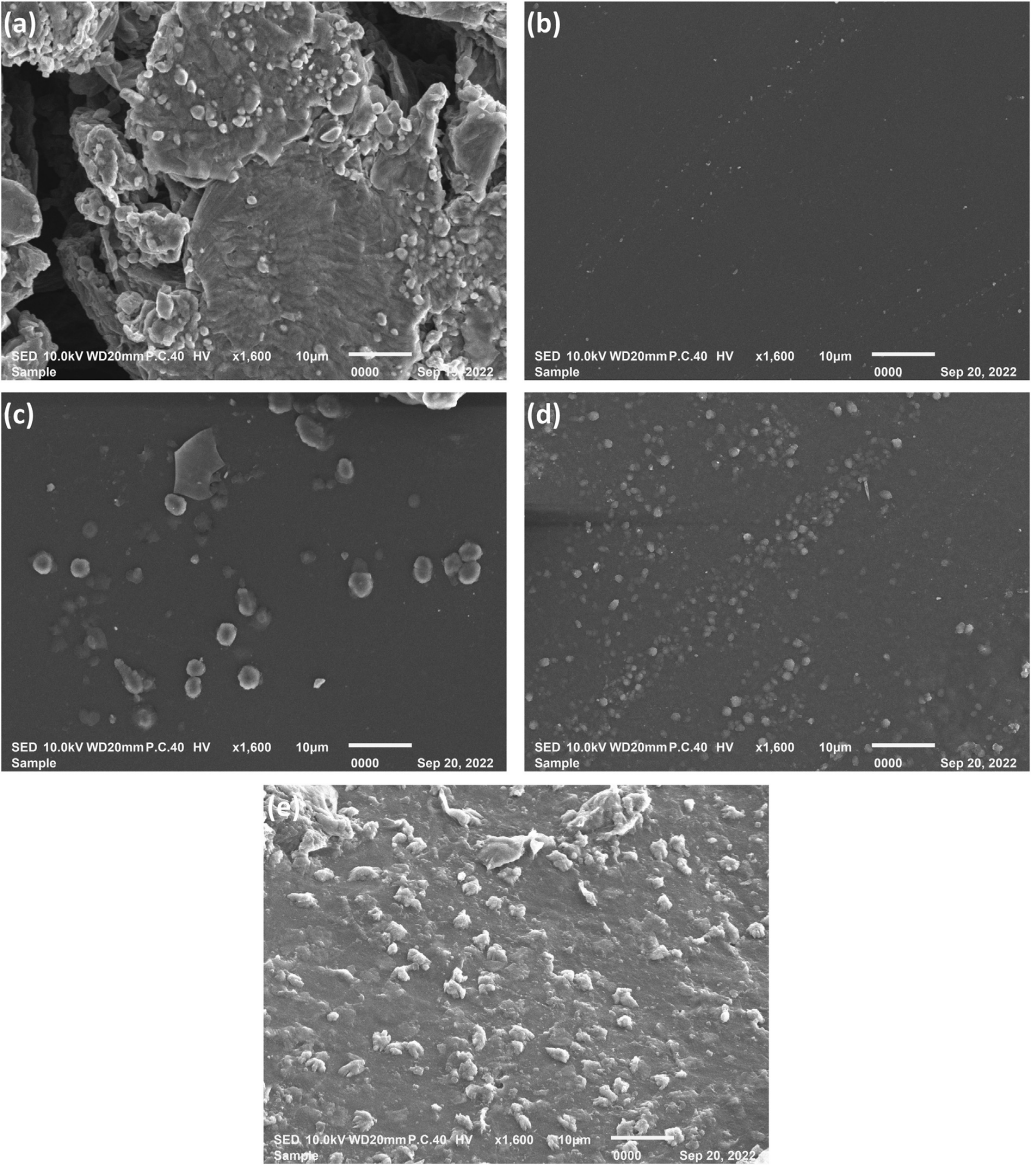
SEM micrographs of (a) GO nanosheets, (b) CTS/PVA polymer blend, (c) blend with 0.3% GO, (d) blend with 0.5% GO, and (e) blend with 1% GO.

As shown in Figure 4a, the synthesized GO had a layered structure associated with ultrathin homogeneous films that were either folded or continuous. The sheets’ edges showed kinked or wrinkled areas. GO particles were sometimes associated and formed aggregates.

#### EDX analysis

According to the elemental analysis of GO mass (Fig. S2, *supplementary data*), an increase in the content of oxygen atoms was observed, because GO is an oxygen rich material with a composition of 55 at% carbon and 44 at% oxygen. The C/O ratio was approximately 1.25, thereby suggesting that a high degree of oxidation by the strong oxidant (KMNO_4_) led to shifts in the distances of GO layers.

### Characterization of prepared CTS/PVA/GO nanocomposite films

#### Macroscopic investigation of CTS/PVA/GO nanocomposite films

In general, CTS/PVA films are yellowish and somewhat translucent because of the PVA. The addition of GO made the films turn black. The intensity of the black color increased with increasing GO amount (Fig. S3, *supplementary data*). The film thickness, measured with digital calipers, was found to be 0.2 mm.

#### XRD analysis

XRD patterns of CTS/PVA and CTS/PVA/GO at different concentrations are shown in Figure 3b. XRD of the CTS/PVA polymer blend showed a sharp diffraction peak at a 2θ of approximately 21°, which was associated with crystallites of PVA. With the addition of GO, the nanocomposite showed identical crystallinity to that of the polymer blend, and the same diffraction peak; this peak showed a slight shift to a lower value when the GO amount was increased.

#### FTIR analysis

FTIR spectra of PVA, CTS, CTS/PVA, and CTS/PVA/GO with different ratios (0.3%, 0.5%, and 1%) are shown in Figure 2. The PVA infrared spectrum (Figure 2b) showed a characteristic diffraction peak at ν 3279 cm^−1^, representing intermolecular H–H bonds and –OH stretching vibrations. The vibrational spectrum detected at ν 2908 cm^−1^ was associated with C–H stretching from the alkyl group. Another absorption peak observed at ν 1086 cm^−1^ corresponded to C–O stretching. The CTS spectrum (Figure 2c) showed a broad band at 3360 cm^−1^, because of the overlapping of –OH and –NH stretching vibrations. The peak at 2868 cm^−1^ corresponded to the stretching vibration of CH_2_. Another band at ν 1590 corresponded to –NH bending (NH_2_). Carbonyl stretching from C–H bending and C–O–C linking were associated with the bands at ν 1376 and 1150 cm^−1^. In the CTS/PVA polymer blend (Figure 2d), the intensity of the ν 3279 cm^−1^ peak decreased to ν 3268 cm^−1^. Additionally, substantial intensity of the absorption band corresponding to C–H stretching vibration was observed at ν 2938 cm^−1^. The peak at ν 1327 cm^−1^ also confirmed the crosslinked structure with PVA, because of the deformation vibration of CH_2_. The peak at ν 1651 cm^−1^ corresponded to C=N stretching, owing to the reaction of the NH_2_ groups of CTS with –OH groups associated with PVA.

In CTS/PVA/GO nanocomposites (Figure 2e), the strong characteristic peak at ν 3268 cm^−1^ was associated with the –OH group present in GO and the stretching of the NH_2_ group in CTS. The peaks at ν 1410 and 1143 cm^−1^ were associated with carboxylate vibrations. The vibrational peak at ν 1643 cm^−1^ was associated with the deformation vibration of absorbed H_2_O molecules. Another characteristic peak at ν 1554 cm^−1^ was associated with the NH_2_ groups of CTS. The peak at ν 2907 cm^−1^ was associated with C–H stretching vibration. The bands at ν 923 and 847 cm^−1^ were associated with the polysaccharide structure present in CTS.

#### SEM investigation

The microstructures of the CTS/PVA polymer blend and nanocomposite films were assessed by SEM (Figure 4). The polymer blend (Figure 4b) showed a smooth surface indicating uniform dispersion and good miscibility between polymers. The surfaces of the pure CTS and pure PVA films were relatively smooth; consequently, the blend was expected to show the same surface morphology. The morphology of the nanocomposite (Figs. 4c, 4d and 4e) showed a rough surface associated with GO dispersion. As the ratio of GO increased, the entire polymer surface was coated by GO, and the surface became rougher and less visible, owing to the substantial interface between GO and the polymer.

#### TGA

**Figure 5:**
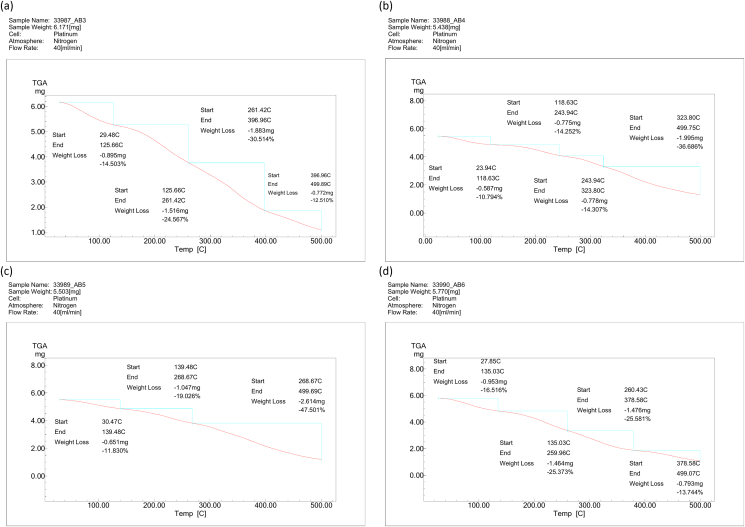
TGA results of CTS/PVA/GO nanocomposite films with different concentrations of GO. (a): CTS/PVA blend with 0% GO, (b) blend with 0.3% GO, (c) blend with 0.5% GO, and (d) blend with 1% GO.

TGA is a valuable method for evaluating polymer stability. As shown in Figure 5, the addition of GO to the polymer blend shifted the degradation to a higher temperature. For the polymer blend (1CTS:1PVA) (Figure 5a), the first degradation step was detected between 29 °C and 125 °C, and a 14% weight loss was associated with breakdown of the polymer backbone. Lower weight loss percentages of 10.7% and 11% in the first degradation step were observed with 0.3% GO (Figure 5b) and 0.5% GO (Figure 5c), respectively. For the polymer blend (1CTS:1PVA) (Figure 5a), the major decomposition temperature ranged from 260 °C to 396 °C, and the weight loss was approximately 30%. Major weight loss occurred at higher temperatures (323–499 °C) with the addition of 0.3–0.5% GO (Figures 5b and 5c, respectively). When the GO loading was increased to 1% (Figure 5d), the major degradation temperature was lower (260–378 °C). An increase in the first degradation step, to 16.5%, was observed with 1% compared with 0.3–0.5% GO. The residue mass was 20% for 1CTS:1PVA, and 26%, 23%, and 21% for 0.3%, 0.5%, and 1% GO dispersions, respectively.

#### Evaluation of mechanical properties

• Tensile strength evaluation

No considerable difference in tensile strength was found between 1CTS (2%):1PVA and 1CTS (3%):1PVA. The addition of GO decreased the tensile strength for different concentrations (0.3%, 0.5%, and 1%), as shown in Table 1.

• Elastic modulus evaluation

**Table 1:** Tensile strength results for the studied groups of CTS-PVA-GO nanocomposite films.

	1CTS (2%): 1PVA	1CTS (2%): 1PVA + 0.3% GO	1CTS (2%):1PVA + 0.5% GO	1CTS (2%): 1PVA +1% GO	1CTS (3%): 1PVA	1CTS (3%): 1PVA + 0.3% GO	1CTS (3%):1PVA + 0.5% GO	1CTS (3%): 1PVA + 1% GO
**Mean (SD)**	8.49 (1.25)	4.17 (0.34)	4.04 (0.83)	6.27 (0.50)	10.00 (0.55)	6.00 (0.56)	8.42 (0.64)	3.77 (0.56)
**Median**	9.40	4.11	4.46	6.10	10.25	6.10	8.50	3.47
**Minimum–maximum**	6.45–9.50	3.54–4.50	2.84–5.05	5.75–7.30	9.40–10.70	5.05–6.75	7.55–9.05	3.06–4.42
***P* value**	**<0.0001**^a^							

a Statistically significant difference at P value ≤ 0.05.

No significant difference was observed in the elastic modulus between 1CTS (2%):1PVA and 1CTS (3%):1PVA. The addition of GO at a concentration of 1% showed a significantly higher elastic modulus for 1CTS (2%):1PVA than 1CTS (2%):1PVA + 0.3% GO and 1CTS (2%):1PVA + 0.5% GO. The addition of GO at 1% concentration to 1CTS (3%):1PVA resulted in a significantly lower elastic modulus than that of 1CTS (3%):1PVA+0.3% GO and 1CTS (3%):1PVA + 0.5% GO (Table 2).

**Table 2:** Elastic modulus results for the studied groups of CTS-PVA-GO nanocomposite films.

	1CTS (2%): 1PVA	1CTS (2%):1PVA + 0.3% GO	1CTS (2%):1PVA + 0.5% GO	1CTS (2%): 1PVA +1% GO	1CTS (3%): 1PVA	1CTS (3%): 1PVA+0.3% GO	1CTS (3%):1PVA + 0.5% GO	1CTS (3%): 1PVA+ 1% GO
**Mean (SD)**	36.83 (17.53)	23.03 (9.60)	13.43 (0.63)	82.92 (12.72)	31.09 (10.00)	34.02 (14.53)	42.53 (8.91)	13.73 (3.50)
**Median**	46.86	26.44	13.71	90.55	30.70	29.98	40.44	13.31
**Minimum–maximum**	13.10–54.07	11.01–33.68	12.31–14.26	60.70–90.78	15.94–46.54	22.78–66.40	27.67–55.88	9.06–20.71
***P* value**	**<0.0001**^a^							

a Statistically significant difference at *P* value ≤ 0.05.

#### In vitro biodegradation assessment

**Figure 6:**
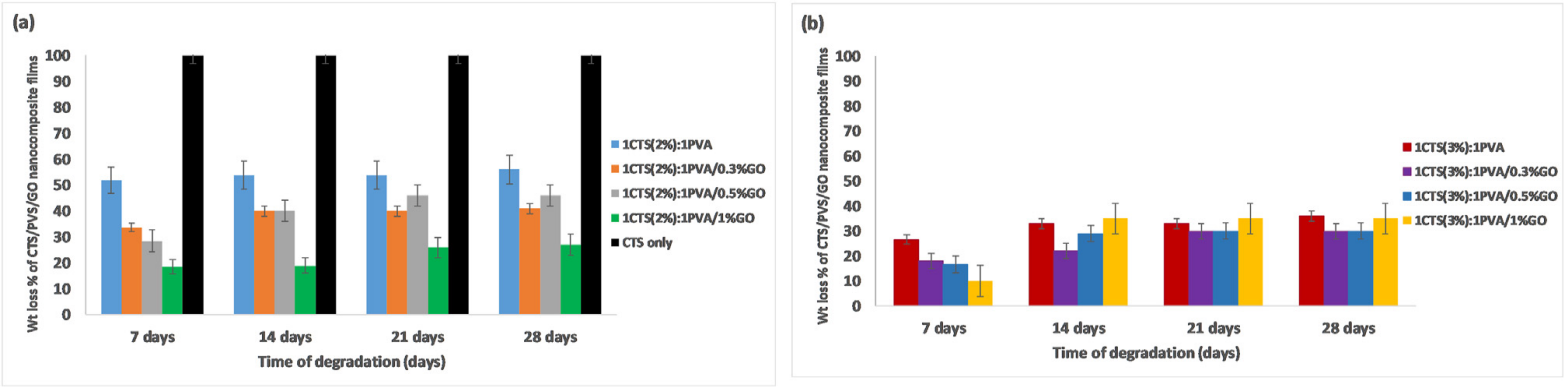
In vitro biodegradation assessment.

As shown in Figure 6, 1CTS (3%):1PVA had a lower degradation rate than 1CTS (2%):1PVA, with total weight losses of approximately 36% and 56%, respectively. The addition of GO to both 1CTS (2%):1PVA and 1CTS (3%):1PVA decreased the degradation rate over 28 days, and the total weight loss also decreased, particularly with higher percentages of GO (1%). Additionally, the early degradation after 7 days decreased after GO addition: the rate of 52% for 1CTS (2%):1PVA changed to 34%, 28%, and 19% after GO addition at concentrations of 0.3%, 0.5%, and 1%, respectively. For 1CTS (3%):1PVA, the early degradation of 30% decreased to 18%, 16%, and 10% after GO addition at concentrations of 0.3%, 0.5%, and 1%, respectively. A pure CTS sample without PVA or GO completely disappeared after 7 days.

#### Swelling study

**Figure 7:**
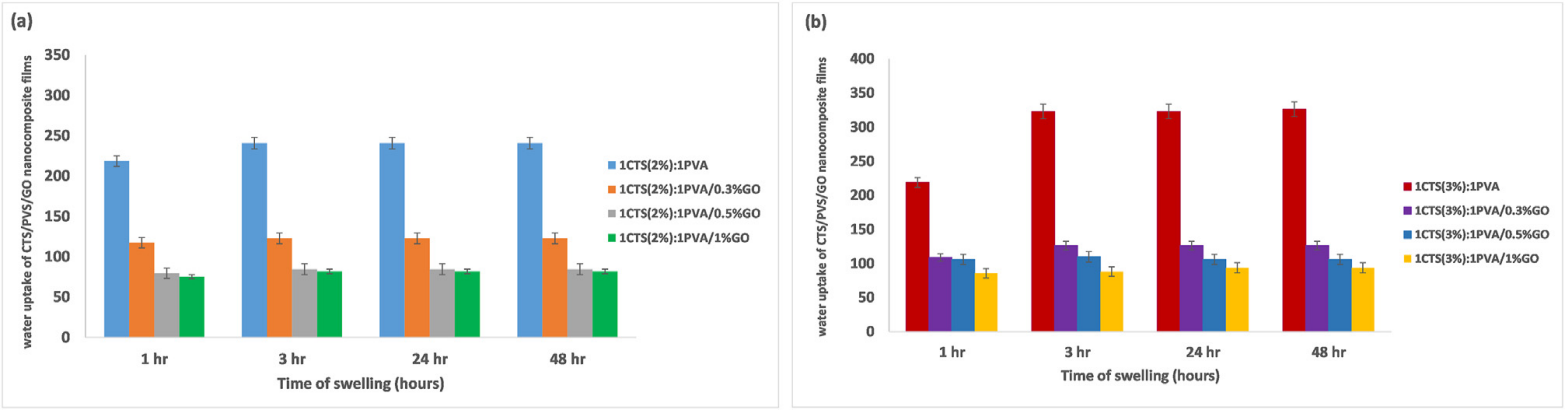
Swelling assessment of CTS/PVA/GO nanocomposite films with different concentrations of GO; CTS/PVA blend with 0% GO, blend with 0.3% GO, blend with 0.5% GO, and blend with 1% GO.

The swelling behavior (Figure 7) showed an increase in water sorption 1 h after immersion in saline; equilibrium was reached after 3 h. Samples that did not contain GO showed greater water sorption. As the concentration of GO increased, swelling decreased. For 1CTS (2%):1PVA and 1CTS (3%):1PVA, the water uptake capacity was greatest (∼240% and 326%). After addition of GO to 1CTS (2%):1PVA, the water uptake capacity decreased to 122%, 84%, and 81% for GO concentrations of 0.3%, 0.5%, and 1%, respectively. The same pattern was observed after GO addition to 1CTS (3%):1PVA, after which the water uptake decreased to 126%, 105%, and 93.6% for GO concentrations of 0.3%, 0.5%, and 1%, respectively.

## Discussion

The production of GO depends on multiple factors including the acid concentration, strength of the oxidizing agent, and decay of the formed intermediate compound. Addition of strong sulfuric acid to graphite leads to intercalation and formation of graphite bisulfate (a graphite intercalated compound). After expansion of the graphene layer, another intercalation occurs at the basal plane.^47^ Amorphization occurs gradually, and the interlayer distance increases. Additionally, the lattice parameter along the c-axis and the number of layers decrease. With addition of KMNO_4_, ultrasonic cleavage of graphite oxide with substantial numbers of surface –OH, –COOH, and epoxy groups occurs. These oxygen functional groups increase the distance between layers and make the structure more hydrophilic.^47^^,^^48^

Raman spectroscopy is used to verify the disorder and weaknesses in the crystal structures of carbonaceous materials. The G band arises from C–C bond stretching. As the disorder of graphite increases, this G band becomes broad at ν 1605 cm^−1^, and a broader D band is observed at ν 1354 cm^−1^. According to Yuan et al. (2017), the presence of broad and intense G and D bands is associated with higher degrees of oxidation than observed in other samples with lower oxidation.^49^ Additionally, the *I*_*D/*_*I*_*G*_ ratio describes the *sp*^*2/*^*sp*^*3*^ carbon ratio, which is associated with the number of change sides made by the functional groups' attachment to carbon, an indicator of the aromatic structure's integrity. A ratio reaching 0.8 indicates structural defects produced by oxidation and attachment of the functional groups.^50^^,^^51^

FTIR characterization of GO revealed the presence of oxygen atoms in the form of –OH, C=O, and C–O, thereby confirming the oxidation process and GO formation. These hydrophilic functional groups are highly important in the compatibility between GO and the polymeric matrix. Additionally, these abundant functional groups have been found to improve GO hydrophilicity.^46^

In the oxidation process of graphite by KMNO_4_, an O-group was added to the structure, thus potentially increasing the distance between layers from 3.4 to 12.4 Å, because of the repulsion forces between layers. According to Absazade,^52^ the oxidation process of graphite leads to narrowing of the graphitic characteristic peak at 2θ = 26.9° and the formation of a new peak at 2θ = 8° which is characteristic of GO. These changes are due to the heterogeneous nature of the oxidized graphite, which contains domains of graphite (*sp*^*2*^) and oxidized graphite (*sp*^*3*^). The oxidation process was also confirmed through elemental analysis by EDX, which yielded results in line with those of Siburian et al.^53^

For preparation of the nanocomposite, CTS was added to PVA, and various GO concentrations (0.3%, 0.5%, and 1%) were added to the polymer blend. The addition of CTS to PVA decreased its crystallinity; consequently, the characteristic peak of PVA in the XRD pattern shifted to a lower value. On the basis of findings from Saeedi et al.,^54^ the CTS characteristic peak was expected to appear at 2θ = ∼20.3°; however, it was not detected, to be confirming the good compatibility between polymers. Additionally, the characteristic peak of GO was not observed, owing to the good dispersion of nanoparticles and their presence in trace amounts.

FTIR analysis of pure PVA revealed a spectrum associated with its hydroxyl groups. The combination of frequencies was produced by the stretching vibrations of the backbone aliphatic CH, CH–OH, and CO. The CTS spectrum showed bands associated with N–H and C–H stretching. For the CTS/PVA polymer blend, the increase in the intensity of C–H stretching indicated good miscibility between PVA and CTS. The lower intensity of the band associated with –OH stretching in the polymer blend than observed for pure PVA might have been due to the vibrational stretching of the –OH of PVA with secondary –NH groups associated with CTS through intermolecular hydrogen bonding. The presence of GO in the blend was indicated by the formation of a hydrogen bond between GO and the polymer. The band at ν 3268 cm^−1^ appeared to increase to ν 3271 cm^−1^, because of the stretching vibration of –OH groups associated with GO.^55^

GO addition to the CTS/PVA blend increased the thermal stability, because the GO provided a physical barrier that caused scaffolds to decompose more slowly with increasing temperature. Furthermore, hydrophilic and electrostatic interactions formed between CTS and GO, and hydrogen interactions formed between the polymer chains and GO, thus limiting the motion of polymer chains, in addition the good dispersion of GO within the polymer blend. As the GO dispersion ratio increased to 1%, the major degradation temperature decreased, and the residue weight was lower (21%) than that with 0.3% GO (26%) and 0.5% GO (23%). These findings were probably due to the aggregation of GO nanoparticles inside the polymer matrix.^56^^,^^57^

The tensile strength of the films decreased with increasing CTS concentration, possibly because excessive CTS prevented the GO sheets from assembling into high-orientation structures; consequently, a substantial number of nanoscale defects limited the materials’ performance. In a previous study, a CTS concentration of 0.3% increased tensile strength after addition of GO, but when the CTS concentration was increased to 0.5% and 1%, the tensile strength sharply decreased.^58^ Because electrostatic interactions are believed to be more powerful than hydrogen bonding, they were considered the main reason for this. Owing to the strong electrostatic interaction between CTS and GO, the addition of excess CTS prevented the formation of a randomly and sparsely distributed structure, and led to precipitation of GO. The orientation of the nanoparticles and their dispersion in the polymeric matrix in a uniform pattern was prevented, thus leading to their aggregation, as previously reported.^59^ An increase in the elastic modulus of 1CTS (2%):1PVA was observed with addition of 1% GO, because GO comprises a two-dimensional sheet of carbon atoms with covalent bonds and different oxygen functional groups, such as hydroxyl, epoxy, and carbonyl groups on the edges and basal planes. Consequently, numerous intermolecular H–H bonds between GO and CTS can form, thereby improving the elastic modulus of the film.^60^ This improvement enables application of the scaffold in hard tissue engineering. The addition of 1% GO in 1CTS (3%):1PVA decreased the elastic modulus of the film, a finding potentially associated with increased heterogenicity of the structure with increasing concentrations of both CTS (3%) and GO (1%). Consequently, aggregation of the nanoparticles rather than uniform dispersion would limit use of the scaffold with high GO concentrations in stress bearing areas, as explained by Mombini et al. in a study using carbon nanotubes to improve the mechanical of CTS/PVA fibers.^61^ CTS degradation occurs through hydrolysis, wherein water molecules interact with polymeric chains and break them into shorter chains. In the body, lysozyme degrades 1–4N-acetyl glucosamines. Amino sugar is released and subsequently removed from the body via the metabolic system. Saccharides are another byproduct of CTS breakdown that travel through metabolic pathways. Incorporation of GO into CTS and PVA blends significantly increased the stability of the films in PBS, and led to lower weight loss and degradation rates with increasing GO concentrations. These findings might have been due to interactions between COOH groups in GO with CTS leading to an increase in crystallinity and decreasing the degradation rate.^62^ The H–H bonding between CTS and PVA explains the stability of the polymer blend, given that the film containing CTS completely degraded only after 7 days.

Finally, the water uptake capacity of the nanocomposite films decreased after GO addition to the polymer matrix. GO addition may prevent water diffusion into the film by decreasing the number of hydrophilic groups such as –COOH and –OH, because hydrogen bonding with the –NH of CTS increases the nanocomposite films' water resistance.^63^ Moreover, the films’ rapid water sorption in the first hour enables them to be used as wound dressings with rapid exudate absorption capacity to avoid exudate accumulation. An equilibrium reached after 3 h may maintain the fluid balance necessary for cellular migration.

## Limitations

The study did not perform biocompatibility testing or investigate the antibacterial action of the prepared scaffold.

## Conclusions

The addition of GO to a CTS/PVA polymeric blend significantly improved the thermal stability of the structure. The residue mass was 20% for (1CTS:1PVA), and 26%, 23%, and 21% for 0.3%, 0.5%, and 1% GO dispersions, respectively. The degradation rate of the nanocomposite decreased with GO addition, particularly for samples containing a high percentage of GO (1%). The water resistance increased with addition of GO to 1CTS (2%):1PVA. The water uptake capacity decreased to 122%–84%, and 81% with GO concentrations of 0.3%, 0.5%, and 1%, respectively. The same pattern was observed after GO addition to 1CTS (3%):1PVA, after which the water uptake decreased to 126%–105% and 93.6%. Regarding mechanical properties, no clear improvement in tensile strength was observed with increasing CTS concentration (2% and 3%), which hindered nanoparticle orientation. The 1CTS (2%):1PVA with 1% GO addition showed a significantly higher elastic modulus than observed with 0.3% and 0.5% GO addition, because of bonding between GO and the polymer matrix.

## Source of funding

This research did not receive any specific grant from funding agencies in the public, commercial or not-forprofit sectors.

## Conflict of interest

The authors declare that they have no competing interests.

## Ethics approval

Not applicable.

## Consent for publication

Not applicable.

## Recommendations

- Biocompatibility and cell viability tests are recommended for the prepared scaffolds.
- Further in vivo testing of the scaffold with different GO concentrations is necessary to detect whether a critical concentration might cause adverse reactions.
- Scaffolds may be loaded with antimicrobial drugs to enhance antimicrobial action; the release properties of the scaffold should be investigated.

## Authors contributions

A. M. Ali: data acquisition, interpretation of data, Formal analysis, and writing the original draft. S. M. Elshabrawy: Conception, design of the work, Formal analysis, draft revision, and supervision. E. A. Kamoun: Design of the work, draft revision, Supervision, and revision of the final draft. All authors approved the manuscript before submission. All authors have critically reviewed and approved the final draft, and are responsible for the content and similarity index of the manuscript.

## Acknowledgments

The authors thank Dr. Hams Hamed for help with statistical analysis. This work was supported by the Deanship of Scientific Research, Vice Presidency for Graduate Studies and Scientific Research, King Faisal University, Saudi Arabia [GRANT NO. ].

## Availability of data and materials

The datasets used and analyzed during the current study are available from the corresponding author on reasonable request.

## References

[1] Stevens M.M. (2008). Biomaterials for bone tissue engineering. Mater Today.

[2] Stevens B., Yang Y., Mohandas A., Stucker B., Nguyen K.T. (2008). A review of materials, fabrication methods, and strategies used to enhance bone regeneration in engineered bone tissues. J Biomed Mater Res B Appl Biomater.

[3] Cao L., Weng W., Chen X., Ding Y., Yan Y., Li H. (2015). Development of degradable and bioactive composite as bone implants by incorporation of mesoporous bioglass into poly (L-lactide). Compos B Eng.

[4] Bose S., Roy M., Bandyopadhyay A. (2012). Recent advances in bone tissue engineering scaffolds. Trends Biotechnol.

[5] Dhandayuthapani B., Yoshida Y., Maekawa T., Kumar D.S. (2011). Polymeric scaffolds in tissue engineering application: a review. Int J Polym Sci.

[6] Surudžić R., Janković A., Bibić N., Vukašinović-Sekulić M., Perić-Grujić A., Mišković-Stanković V. (2016). Physico–chemical and mechanical properties and antibacterial activity of silver/poly (vinyl alcohol)/graphene nanocomposites obtained by electrochemical method. Compos B Eng.

[7] Aslam M., Kalyar M.A., Raza Z.A. (2017). Fabrication of reduced graphene oxide nanosheets doped PVA composite films for tailoring their opto-mechanical properties. Appl Phys A.

[8] Vilar Junior J.C., Ribeaux D.R., Alves da Silva C.A., Campos-Takaki D., Maria G. (2016). Physicochemical and antibacterial properties of chitosan extracted from waste shrimp shells. Internet J Microbiol.

[9] Anitha A., Sowmya S., Kumar P.T.S., Deepthi S., Chennazhi K.P., Ehrlich H. (2014). Chitin and chitosan in selected biomedical applications. Prog Polym Sci.

[10] Muxika A., Etxabide A., Uranga J., Guerrero P., de La Caba K. (2017). Chitosan as a bioactive polymer: processing, properties and applications. Int J Biol Macromol.

[11] Aranaz I., Alcántara A.R., Civera M.C., Arias C., Elorza B., Heras Caballero A. (2021). Chitosan: an overview of its properties and applications. Polymers (Basel).

[12] Ke C.L., Deng F.S., Chuang C.Y., Lin C.H. (2021). Antimicrobial actions and applications of chitosan. Polymers (Basel).

[13] Kim I.Y., Seo S.J., Moon H.S., Yoo M.K., Park I.Y., Kim B.C. (2008). Chitosan and its derivatives for tissue engineering applications. Biotechnol Adv.

[14] Chen S., Wang H., Jian Z., Fei G., Qian W., Luo G. (2020). Novel poly (vinyl alcohol)/chitosan/modified graphene oxide biocomposite for wound dressing application. Macromol Biosci.

[15] Kamoun E.A., Kenawy E.R.S., Chen X. (2017). A review on polymeric hydrogel membranes for wound dressing applications: PVA-based hydrogel dressings. J Adv Res.

[16] Chetouani A., Elkolli M., Bounekhel M., Benachour D. (2017). Chitosan/oxidized pectin/PVA blend film: mechanical and biological properties. Polym Bull.

[17] Rodríguez-Rodríguez R., Espinosa-Andrews H., Velasquillo-Martínez C., García-Carvajal Z.Y. (2020). Composite hydrogels based on gelatin, chitosan and polyvinyl alcohol to biomedical applications: a review. Int J Polym Mater.

[18] Agrawal P., Pramanik K. (2016). Chitosan-poly (vinyl alcohol) nanofibers by free surface electrospinning for tissue engineering applications. Tissue Eng Regen Med.

[19] Farzinfar E., Paydayesh A. (2019). Investigation of polyvinyl alcohol nanocomposite hydrogels containing chitosan nanoparticles as wound dressing. Int J Polym Mater.

[20] Cui Z., Zheng Z., Lin L., Si J., Wang Q., Peng X. (2018). Electrospinning and crosslinking of polyvinyl alcohol/chitosan composite nanofiber for transdermal drug delivery. Adv Polym Technol.

[21] Ge C., Lao F., Li W., Li Y., Chen C., Qiu Y. (2008). Quantitative analysis of metal impurities in carbon nanotubes: efficacy of different pretreatment protocols for ICPMS spectroscopy. Anal Chem.

[22] Sivashankari P.R., Prabaharan M., Maiti S., Jana S. (2019). Polysaccharide carriers for drug delivery.

[23] Yu P., Bao R.Y., Shi X.J., Yang W., Yang M.B. (2017). Self-assembled high-strength hydroxyapatite/graphene oxide/chitosan composite hydrogel for bone tissue engineering. Carbohydr Polym.

[24] Xiong S., Luo J., Wang Q., Li Z., Li J., Liu Q. (2021). Targeted graphene oxide for drug delivery as a therapeutic nanoplatform against Parkinson's disease. Biomater Sci.

[25] Lee S.Y., Kwon M., Raja I.S., Molkenova A., Han D.W., Kim K.S., Han D., Hong S.W. (2022). Multifaceted biomedical applications of graphene.

[26] Zhihui K., Min D. (2022). Application of graphene oxide-based hydrogels in bone tissue engineering. ACS Biomater Sci Eng.

[27] Wei P., Wang L., Xie F., Cai J. (2022). Strong and tough cellulose–graphene oxide composite hydrogels by multi-modulus components strategy as photothermal antibacterial platform. J Chem Eng.

[28] Manousi N., Rosenberg E., Deliyanni E.A., Zachariadis G.A. (2020). Sample preparation using graphene-oxide-derived nanomaterials for the extraction of metals. Molecules.

[29] Mazaheri M., Akhavan O., Simchi A. (2014). Flexible bactericidal graphene oxide–chitosan layers for stem cell proliferation. Appl Surf Sci.

[30] Sohail M., Saleem M., Ullah S., Saeed N., Afridi A., Khan M. (2017). Modified and improved Hummer's synthesis of graphene oxide for capacitors applications. Mod Electron Mater.

[31] Venkataprasanna K.S., Prakash J., Vignesh S., Bharath G., Venkatesan M., Banat F. (2020). Fabrication of Chitosan/PVA/GO/CuO patch for potential wound healing application. Int J Biol Macromol.

[32] He L., Dumée L.F., Feng C., Velleman L., Reis R., She F. (2015). Promoted water transport across graphene oxide–poly (amide) thin film composite membranes and their antibacterial activity. Desalination.

[33] Kurapati R., Vaidyanathan M., Raichur A.M. (2016). Synergistic photothermal anti-microbial therapy using graphene oxide/polymer composite layer-by-layer thin films. RSC Adv.

[34] Li P., Sun S., Dong A., Hao Y., Shi S., Sun Z. (2015). Developing of a novel antibacterial agent by functionalization of graphene oxide with guanidine polymer with enhanced antibacterial activity. Appl Surf Sci.

[35] Liao K.H., Lin Y.S., Macosko C.W., Haynes C.L. (2011). Cytotoxicity of graphene oxide and graphene in human erythrocytes and skin fibroblasts. ACS Appl Mater Interfaces.

[36] Ruiz O.N., Fernando K.A.S., Wang B., Brown N.A., Luo P.G., McNamara N.D. (2011). Graphene oxide: a nonspecific enhancer of cellular growth. ACS Nano.

[37] Srimaneepong V., Skallevold H.E., Khurshid Z., Zafar M.S., Rokaya D., Sapkota J. (2022). Graphene for antimicrobial and coating application. Int J Mol Sci.

[38] Unnithan A.R., Park C.H., Kim C.S. (2016). Nanoengineered bioactive 3D composite scaffold: a unique combination of graphene oxide and nanotopography for tissue engineering applications. Compos B Eng.

[39] Cao L., Zhang F., Wang Q., Wu X. (2017). Fabrication of chitosan/graphene oxide polymer nanofiber and its biocompatibility for cartilage tissue engineering. Mater Sci Eng C.

[40] Krishnan S.K., Singh E., Singh P., Meyyappan M., Nalwa H.S. (2019). A review on graphene-based nanocomposites for electrochemical and fluorescent biosensors. RSC Adv.

[41] Yenier Z., Seki Y., Şen İ., Sever K., Mermer Ö., Sarikanat M. (2016). Manufacturing and mechanical, thermal and electrical characterization of graphene loaded chitosan composites. Compos B Eng.

[42] Rosner B. (2015).

[43] Abd-Elhamid A.I., Kamoun E.A., El-Shanshory A.A., Soliman H.M.A., Aly H.F. (2019). Evaluation of graphene oxide-activated carbon as effective composite adsorbent toward the removal of cationic dyes: composite preparation, characterization, and adsorption parameters. J Mol Liq.

[44] Tavakoli M., Karbasi S., Bakhtiari S.S.E. (2019). Evaluation of physical, mechanical, and biodegradation of chitosan/graphene oxide composite as bone substitutes. Polym-Plast Tech Mat.

[45] Pandele A.M., Ionita M., Crica L., Vasile E., Iovu H. (2017). Novel Chitosan-poly (vinyl alcohol)/graphene oxide biocomposites 3D porous scaffolds. Compos B Eng.

[46] Yadav M., Ahmad S. (2015). Montmorillonite/graphene oxide/chitosan composite: synthesis, characterization and properties. Int J Biol Macromol.

[47] Aliyev E., Filiz V., Khan M.M., Lee Y.J., Abetz C., Abetz V. (2019). Structural characterization of graphene oxide: surface functional groups and fractionated oxidative debris. Nanomaterials.

[48] Bîru E.I., Iovu H., do Nascimento G.M. (2018). Raman spectroscopy.

[49] Yuan R., Yuan J., Wu Y., Chen L., Zhou H., Chen J. (2017). Efficient synthesis of graphene oxide and the mechanisms of oxidation and exfoliation. Appl Surf Sci.

[50] Abd-Elhamid A.I., Aly H.F., Soliman H.A.M., El-Shanshory A.A. (2018). Graphene oxide: follow the oxidation mechanism and its application in water treatment. J Mol Liq.

[51] Rokaya D., Srimaneepong V., Qin J., Siraleartmukul K., Siriwongrungson V. (2019). ‏ graphene oxide/silver nanoparticle coating produced by electrophoretic deposition improved the mechanical and tribological properties of NiTi alloy for biomedical applications. JNN.

[52] Abaszade R.G. (2022). Synthesis and analysis of flakes graphene oxide. J Optoelectron Biomed Mater.

[53] Siburian R., Sihotang H., Raja S.L., Supeno M., Simanjuntak C. (2018). New route to synthesize of graphene nano sheets. Orient J Chem.

[54] Saeedi F., Montazeri A., Bahari Y., Pishvaei M., Jannat B. (2021). A study on the viscoelastic behavior of chitosan-polyvinyl alcohol-graphene oxide nanocomposite films as a wound dressing. Polym Polym Compos.

[55] Das L., Das P., Bhowal A., Bhattachariee C. (2020). Synthesis of hybrid hydrogel nano-polymer composite using Graphene oxide, Chitosan and PVA and its application in waste water treatment. Environ Technol Innov.

[56] Pandele A.M., Ionita M., Crica L., Dinescu S., Costache M., Iovu H. (2014). Synthesis, characterization, and in vitro studies of graphene oxide/chitosan–polyvinyl alcohol films. Carbohydr Polym.

[57] Montazeri A., Saeedi F., Bahari Y., Ahmadi Daryakenari A. (2021). Preclinical assessment of chitosan–polyvinyl alcohol–graphene oxide nanocomposite scaffolds as a wound dressing. Polym Polym Compos.

[58] Geng L., Lin Y., Chen S., Shi S., Cai Y., Li L. (2020). Superior strength and toughness of graphene/chitosan fibers reinforced by interfacial complexation. Compos Sci Technol.

[59] Qi C., Zhao L., Lin Y., Wu D. (2018). Graphene oxide/chitosan sponge as a novel filtering material for the removal of dye from water. J Colloid Interface Sci.

[60] Ma J., Liu C., Li R., Wang J. (2012). Properties and structural characterization of chitosan/poly (vinyl alcohol)/graphene oxide nano composites. E-Polymers.

[61] Mombini S., Mohammadnejad J., Bakhshandeh B., Narmani A., Nourmohammadi J., Vahdat S. (2019). Chitosan-PVA-CNT nanofibers as electrically conductive scaffolds for cardiovascular tissue engineering. Int J Biol Macromol.

[62] Khan M.U.A., Yaqoob Z., Ansari M.N.M., Razak S.I.A., Raza M.A., Sajjad A. (2021). Chitosan/poly vinyl alcohol/graphene oxide based pH-responsive composite hydrogel films: drug release, anti-microbial and cell viability studies. Polymers (Basel).

[63] Ying Y., Wu Y., Huang J. (2020). Preparation and characterization of chitosan/poly (vinyl alcohol)/graphene oxide films and studies on their antibiofilm formation activity. J Biomed Mater Res A.

