# Supplementary material for: Evaluation of the mechanical properties and degradation behavior of chitosan-PVA-graphene oxide nanocomposite scaffolds in vitro
Source: J Taibah Univ Med Sci. 2024 Apr 30;19(3):585–97. doi: 10.1016/j.jtumed.2024.04.008 (PMC11090909; doi:10.1016/j.jtumed.2024.04.008)
Supplement: Multimedia component 1 [file mmc1.docx]

**Supplementary Data**

**Evaluation of mechanical properties and biodegradation behavior of chitosan- polyvinyl alcohol-graphene oxide nanocomposite scaffolds: *In-vitro* study for dental applications**

Asmaa M. Ali^a^ M.Sc., Sonia M. Elshabrawy^b^ Ph.D., Elbadawy A. Kamoun^c,d,*^ Ph.D.

^a^ Dental Biomaterial Department, Faculty of Dentistry, Kafr El-Sheikh University, Egypt.

^b^ Dental Biomaterials Department, Faculty of Dentistry, Alexandria University, Alexandria 21512, Egypt.

^c^ Department of Chemistry, College of Science, King Faisal University, Al-Ahsa 31982, Saudi Arabia.

^d^ Polymeric Materials Research Department, Advanced Technology and New Materials Research Institute (ATNMRI), City of Scientific Research and Technological Applications (SRTA-City), New Borg Al-Arab City, Alexandria 21934, Egypt.

****Corresponding author:*** *E.A. Kamoun, E-mail:* [*ekamoun@kfu.edu.sa*](mailto:ekamoun@kfu.edu.sa)*,* [*badawykamoun@yahoo.com*](mailto:badawykamoun@yahoo.com)*; Tel: +20-1283320302.*


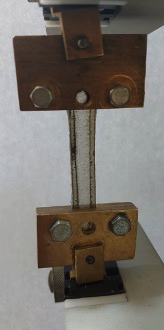


**Figure S1**: Tensile strength evaluation of CTS-PVA-GO nanocomposite films.


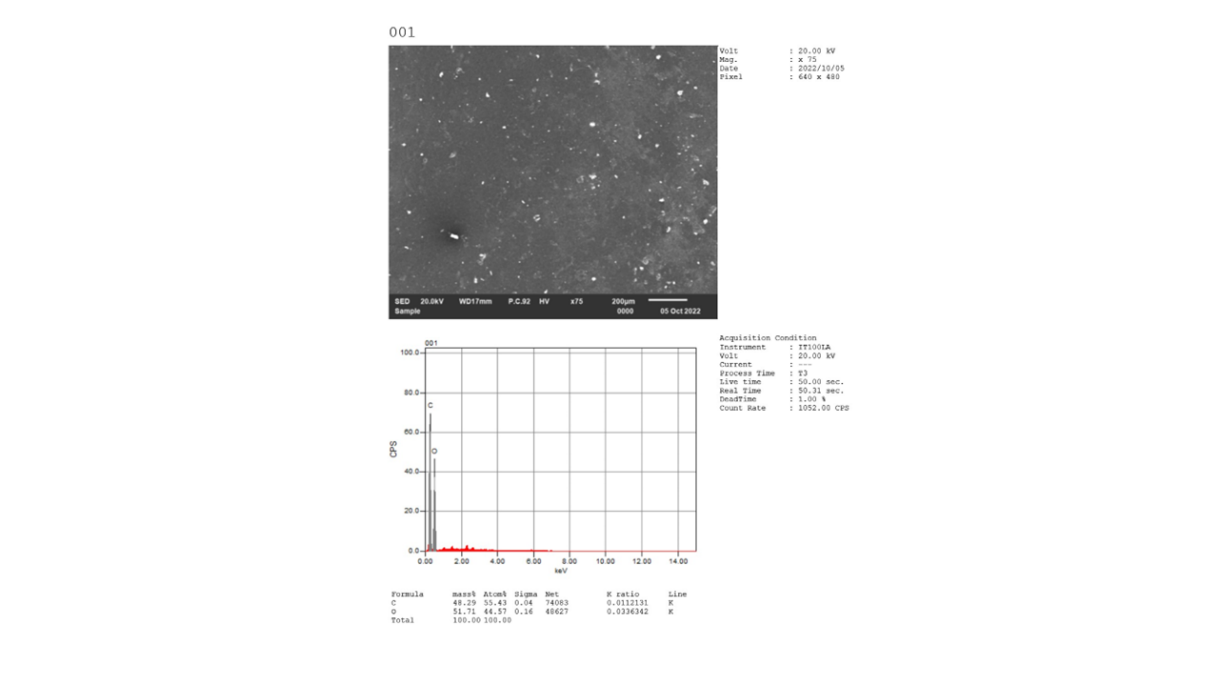
**EDX analysis of GO nanosheets**

**Figure S2**: EDX analysis of GO nan sheets.

**Macroscopic investigation of the nanocomposite films:**


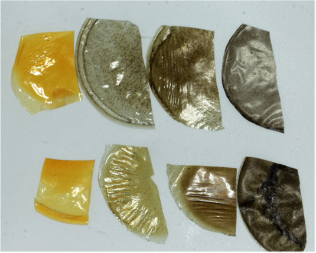


**Figure S3**: macroscopic image of the nanocomposite films, upper from left: 1CTS 2%:1PVA then with 0.3%,0.5% and 1% GO; lower from left: 1CTS 3%:1PVA then with 0.3%,0.5% and 1% GO.
